# Performance of Ultrasound in the Clinical Evaluation of Gout and Hyperuricemia

**DOI:** 10.1155/2021/5550626

**Published:** 2021-04-05

**Authors:** Ling Cao, Tianyi Zhao, Chunmei Xie, Shucong Zheng, Weiguo Wan, Hejian Zou, Xiaoxia Zhu

**Affiliations:** ^1^Division of Rheumatology, Huashan Hospital, Fudan University, Shanghai, China 200040; ^2^Institute of Rheumatology, Immunology, and Allergy, Fudan University, Shanghai, China 200040; ^3^Ultrasonic Department, Huashan Hospital North, Fudan University, Shanghai, China 200040

## Abstract

**Objective:**

To evaluate monosodium urate (MSU) crystal deposition and related lesions in the joints of patients with gout and hyperuricemia (HUA) using ultrasound. To explore the association between ultrasound findings and clinical features in gout and HUA.

**Methods:**

A total of 202 patients with gout and 43 asymptomatic patients with HUA were included. The clinical data and ultrasonic assessment results were collected and statistically analyzed.

**Results:**

Deposition of MSU crystals was found in 25.58% (11/43) of patients with asymptomatic HUA and 76.24% (154/202) of patients with gout. Of the 1,082 joints from patients with gout examined, 33.09% (358/1082) displayed MSU crystal deposition. In the joints with MSU crystal deposition, 77.37% (277/358) had a history of attacks. Among the joints of gouty arthritis, double contour sign (DCS), hyperechoic aggregate (HAG), and tophi were found in 32.65% (159/487), 7.80% (38/487), and 24.64% (120/487) of the joints, respectively. DCS and tophi, but not HAG, increasingly appeared with the extension of gout duration. In patients with more than 15 years of gout history, DCS, Tophi, and HAG were found in 48.18%, 40.00%, and 6.36% of US assessed joints, respectively. In patients with gout, synovial lesion and bone erosion were found in 17.74% (192/1082) and 7.58% (82/1082) of joints, respectively. The synovial lesion was related to HAG, while bone erosion was related to tophi and DCS. Nephrolithiasis was detected in 20.30% (41/202) of patients with gout and 4.65% (2/43) of HUA patients, indicating that nephrolithiasis occurred in more patients with gout than in patients with HUA.

**Conclusion:**

HAG is an early performance of MSU crystal deposition in joints of gout and HUA. Both DCS and tophi are risk factors for bone erosion. Early urate-lowering therapy (ULT) should be considered in patients with gout, DCS, or tophi.

## 1. Introduction

Gout is a common inflammatory disease induced by the deposition of monosodium urate (MSU) crystals in joints and surrounding soft tissues, and hyperuricemia (HUA) is a critical factor for developing symptomatic gout. Most HUA patients do not have gouty arthritis, although MUS crystals are detected in their joints [1, 2]. Chronic pain, soreness, or numbness in the joints is reported in some patients without convincing clinical evidence of a gouty attack, and it is difficult to differentiate gout from osteoarthritis or other chronic arthritis. Noninvasive imaging evidence of urate deposition in joints is valuable and helpful for differential diagnosis [3].

Ultrasound (US), a noninvasive, free of ionizing radiation, convenient, and inexpensive approach, has recently been used to identify MSU crystal deposits for diagnosing gout [4, 5]. A standardized definition of ultrasound lesions with the elementary morphostructural changes in gout has been established in an international consensus [6]. Double contour sign (DCS), hyperechoic aggregates (HAG), and tophi are considered characteristic US abnormalities of MSU crystal deposits. Furthermore, synovial lesions (i.e., synovial hypertrophy and synovitis) and bone erosion are regularly detected by ultrasound in gout and HUA patients [2].

US findings are known to be crucial evidence for the diagnosis or differential diagnosis for gout [7, 8]. The various US phenomena may indicate various joint injuries. Tophi detected by US is reported to be associated with worse foot pain and disability [9]. However, US evidence of MSU crystal deposition can be found in asymptomatic joints in gouty patients, while MSU crystals may not always be detected in the gouty joints with attacks [10]. Furthermore, joint US may show the sign of MSU crystal deposition in asymptomatic HUA and normal people [11]. These findings suggest that a reliable correlation between MSU crystal deposition and gouty attack or erosion remains unclear.

In this study, we retrospectively analyzed the joints' US results from patients who were diagnosed with gout or HUA to validate the US assessment of MSU crystal deposition and lesion in joints and evaluate the clinical value of US application in the diagnosis and prevention of gout and HUA.

## 2. Methods

### 2.1. Study Cohort and Methods

Patients aged 18–75 years, diagnosed with gout or asymptomatic HUA in a gout-specialized clinic in Huashan Hospital from Aug 1^st^, 2016, to Feb 28^th^, 2019, were eligible for this study. A total of 245 patients (202 with gout, 43 with asymptomatic HUA) were included. The required inclusion criteria for patients with gout matched the gout classification criteria of the American College of Rheumatology (ACR)/European League Against Rheumatism (EULAR) (2015). The exclusion criteria for patients with gout were prior diagnosis of other crystal-related arthropathies, such as calcium pyrophosphate deposition disease with visible tophi. Urate levels in fasting serum in all 43 HUA patients were greater than 420 *μ*mol/L, and this was confirmed at least twice. Besides the US results, all the following data, including demographics (i.e., sex, age, and disease duration), body mass index (BMI), and the clinical features of affected joints, were collected.

In the 202 patients with gout, 1,082 joints, including the first metatarsophalangeal joint (MTP1), ankle, knee, acrotarsium, elbow, wrist, and hand joints, were analyzed using US. In the 43 HUA patients, 256 joints were examined. Most of the joints were the three vulnerable pairs of lower joints (MTP1, ankle, and knee), and the others were the joints with mild clinical manifestation, including numb, slight pain, or discomfort. The rheumatologist decided which and how many joints underwent US examination based on clinical judgment (the joints with symptoms and potentially affected joints).

Ethical approval for the study was obtained from the Institutional Review Board for Human Studies at Huashan Hospital, Fudan University (approval number: 2012137). All the included patients had provided a fully informed written consent form before data collection.

### 2.2. US Assessment

The US examination was performed by skilled sonographers who had more than 10 years of experience in the musculoskeletal US in Huashan Hospital. Aplio i900 color ultrasonic diagnostic apparatus (probe frequency 5–18 MHz) was used for US examination. According to the international consensus of the standardized definition of US gout lesion published by OMERACT (Outcome Measures in Rheumatology), US Gout Task Force was employed [6, 12], and MSU crystal deposition in joints was diagnosed based on DCS, hyperechoic aggregates (HAG), and tophi. Synovial hypertrophy, synovitis, and bone erosion were considered joint damage.

### 2.3. Statistical Analysis

The analysis was performed using SPSS 19.0 (IBM). All data were presented as mean ± SD or proportions. Comparisons of baseline data between the two groups were tested for statistical significance using a *t-*test or one-way analysis of variance test with a least significant difference multiple comparison test. *p* values <0.05 were considered significant. Pearson's correlation coefficient was calculated to examine correlations between variables.

## 3. Results

### 3.1. Demographics and Clinical Characteristics of the Study Population

Demographics and clinical characteristics are summarized in Table 1. Among the 202 patients with gout, 191 (94.55%) were male. The average age was 46.90 years old. Among the 43 HUA patients, 31 (72.09%) were male. The average age was 44.47 years old. BMI in both gout (25.59 ± 3.48) and HUA (25.64 ± 3.03) patients was higher than the normal value (18.5–23.9), and there was no significant difference between the two groups of patients. Serum uric acid (SUA) in patients with gout (524.24 ± 79.68) was higher than in HUA patients (493.40 ± 66.85), which might be due to the longer course of HUA in the gout group.

The urate crystal deposits in the kidney directly result in chronic urate nephropathy. In Table 1, the creatinine clearance rate (Ccr) in patients with gout (95.55 ± 2.55 mL/minute) is lower than in HUA patients (106.42 ± 5.54 mL/minute). Ccr < 80 ml/minute was detected in 39.60% (80/202) of patients with gout and 20.93% (9/43) of HUA patients. Hyperlipidemia and hyperglycemia were known to coexist with HUA and gout. In our study, both fasting blood glucose (GLT) and triglyceride (TG) were found to be higher in patients with gout than in HUA patients (Table 1). In patients with gout, our results showed more common comorbidities, such as kidney damage and metabolic disorder.

### 3.2. Clinical Characteristics of Gouty Attacks in the Population with Gout

**Figure 1:**
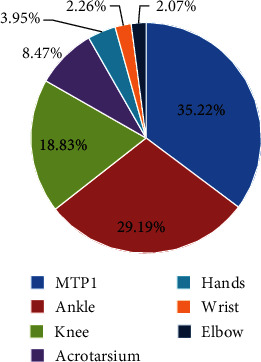
Clinical characteristics of gouty attacks in the gout population.

We further analyzed the relationship between ultrasound abnormalities signs and clinical manifestations in the gout patients (Table 2, Figure 1). There were a total of 531 joints, including 187 (35.22%) of MTP1, 155 (29.19%) of ankles, 100 (18.83%) of knees, 45 (8.47%) of acrotarsium, 21 (3.95%) of hand joints, 12 (2.26%) of the wrist, and 11 (2.07%) of the elbow in the 202 patients who had clinical attacks. More right joints showed attacks than left ones, although there was no statistical significance. Of the 202, the headmost involved joints were 99 (49.01%) on MTP1, 61 (31.20%) on the ankle, 21(10.40%) on acrotarsium, 14 (6.93%) on the knee, 3 (1.49%) on the wrist, and 4 (1.98%) on hand joints. The results indicate that MTP1, ankle, and acrotarsium are more susceptible to be attacked.

### 3.3. Global US Findings in Patients with Gout and Hyperuricemia

In the 202 patients with gout, MSU crystals were detected in at least one of the joints in 76.24% (154/202), and 23.76% (48/202) of patients did not present any MSU crystals in the examined joints. MSU crystals were detected in 358 (33.09%) joints of the 1,082 joints examined (Table 3(a)). Two-hundred and seventy-seven (77.37%) of the 358 joints displayed MSU crystals and attacks. In the HUA patients, MSU crystals were detected in 11 joints among the 256 joints that underwent US examination, and the positive rate was 4.3% (11/256). Interestingly, these 11 joints belonged to 11 patients, and each patient had only one joint with positive US signs of MSU crystal deposition (Table 3(a)).

In the 1,082 joints of the patients with gout, 487 joints had at least one attack, while no attacks were reported in 541 joints (Table 3(b)). In the 487 joints, MSU crystals were found in 56.88% (277/487) using US. Among these joints, 32.65% (159/487), 7.80% (38/487), and 24.64% (120/487) were DCS, HAG, and tophi, respectively.

### 3.4. Synovial Lesion and Bone Erosion in the Patients with Gout

Besides the US signs of MSU crystal deposition, synovial lesions (i.e., synovial hypertrophy and synovitis) and bone erosion were regularly detected in the patients. In the patients with gout, synovial lesions were found in 192 (17.74%) joints, and bone erosion was found in 82 (7.58%) joints in 1,082 joints examined. In the 192 joints with synovial lesions, 24.48% (47/192), 11.98% (23/192), and 12.50% (24/192) were simultaneously positive for DCS, HAG, and tophi, respectively. In the 82 joints with bone erosion, 56.10% (46/82), 7.69% (4/82), and 75.61% (62/82) were simultaneously positive for DCS, HAG, and tophi, respectively (Table 4(a)). We further analyzed the correlation between synovial lesions and bone erosion with three US assessments. The results showed that synovial lesions were associated with HAG (*p* < 0.01) (Table 4(b)), and bone erosion was associated with tophi (*p* < 0.001) and DCS (*p* < 0.01) (Table 4(c)).

### 3.5. Course Time and the MSU Crystals in Patients with Gout

The progression of gout can be defined by four pathophysiological stages: hyperuricaemia without evidence of monosodium urate crystal deposition or gout, crystal deposition without symptomatic gout, crystal deposition with acute gout flares, and advanced gout characterized by tophi, chronic gouty arthritis, and radiographic erosions. When the urate level in serum exceeds its saturation concentration, the precipitated urate crystals deposit in joints and soft tissues. In this study, we found that MSU crystal deposition was correlated with the SUA level (*p* < 0.01) and the duration (*p* < 0.01). Furthermore, many patients had no awareness of their SUA level before seeking clinical specialists. In 202 patients with gout, only 17.33% (35/202) of the patients came for treatment at their early stage (course < 1 year). Most had more than one year of gout history (Table 5(a)).

In patients with gout, the proportion of US-positive signs of MSU crystal deposition gradually increased, especially DCS and tophi during gout. In patients with more than 15 years of gout history, DCS and tophi were detected in 48.18% and 40.00% of joints, respectively, whereas in patients with less than one year of gout history, DCS and tophi were found only in 6.29% and 5.03% of joints, respectively. HAG showed no notable rising as gout duration extension. HAG was found in 5.03% (8/159) of joints of the patients who had less than one year of gout course, and it was 6.36% (7/110) in patients with more than 15 years of gout course (Table 5(a)).

In 35 patients with an early stage (gout course was less than one year), 28 patients came to the clinic at their first gout attack, and the affected joints were MTP1 [13], ankles [6], knee [1], acrotarsium [2], and hand joints [2]. In the 17 affected MTP1 joints, eight (47.06%) were positive for MSU crystal deposition (3 DCS, 3 HAG, 2 tophi, and 1 DCS + tophi) (Table 5(b)).

### 3.6. Correlation of Joint MSU Crystal Deposition with Nephrolithiasis

Patients with gout were reported to be prone to have nephrolithiasis, acute renal colic, and hematuria, although it is difficult to determine the type of crystal in the kidney. In this study, we further analyzed the US data of the kidney. Nephrolithiasis was defined as US signs of calculus or crystal deposition in the kidney. We found that nephrolithiasis was detected in 20.30% (41/202) of patients with gout and 4.65% (2/43) of HUA patients, indicating that nephrolithiasis occurred more commonly in patients with gout than in hyperuricemia patients. The findings also showed that nephrolithiasis was remarkably relevant to MSU crystal deposition in joints in (*p* < 0.05) (Table 6).

## 4. Discussion

The demographics of cohorts in this study show that the average ages of gout and HUA patients in this study were 46.90 and 44.47 years old, respectively, which were younger than the previously reported average age of 52.69 years from the HUA data in a Chinese national cross-sectional survey in 2014 [14]. This suggests that gout and HUA are increasing in the younger population due to lifestyle changes [15]. Our results are consistent with previous reports that high BMI, hyperlipidemia, and hyperglycemia are notably complicated with gout and HUA, especially gout [16]. Therefore, middle-aged males with certain metabolic syndromes should be listed in the attention-demanding high-risk population of gout and HUA.

Our data indicate that lower limb joints are more vulnerable in patients with gout. MTP1 (49.01%), followed by the ankle (31.20%) and acrotarsium (10.40%), is the most affected joints. The upper limb joints, including hands, wrist, and elbow, are rarely involved. This result strongly supports the gout diagnostic value of MTP1 and ankle attacks, especially at an early stage.

For patients without classic symptoms, MSU crystal deposition could be identified by image-based examination, such as dual-energy computed tomography (DECT) and US. It has been reported that the MSU burden volume, which is predictive of the risk of flares [17], can be measured using DECT [13]. Due to being noninvasive, free of ionizing radiation, convenient, and inexpensive, US is more widely used in the clinic. Our study shows that MSU deposition can be detected by US, and the most frequently affected joint is MTP1 in the gout patients. This result is consistent with clinical findings reported by other groups [7].

Previous research suggested that MSU deposition in joints is a crucial factor of gouty arthritis attack [4]. In this study, 28 gout patients came to outpatients at their first acute attack and took a joint examination by US. In the 28 patients, only 39.29% (11/28) attacked joints were found with MSU deposition. Furthermore, among the total 11 gouty joints with initial attacks, large joints such as the knee and ankle were the majority of MSU deposition-positive joints. Only one MTP1 was positive of MSU crystal deposition detected by US.

Moreover, of the 17 patients who were most attacked on MTP1 at an early stage (course < 1 year), 47.06% (8/17) were positive for MSU crystal deposition in the MTP1. These results raise a serious question about how sensitive the US is to detect MSU crystal deposition in joints, particularly in smaller joints of the lower extremities at an early stage. Surprisingly, 77.37% of the patients with US-detected MSU crystal deposits in joints had gouty attacks in the past. Due to practical reasons, the selection of joints for US examination is based on rheumatologist personal judgment in this retrospective study, and a certain bias cannot be ruled out. This interpretation is consistent with previous research, as ultrasound features of MSU crystal deposition had a high positive predictive value but more limited sensitivity for early gout [18]. Further investigation is warranted to validate the US's sensitivity and specificity in detecting MSU crystal deposition in various joints.

According to “2018 updated European League Against Rheumatism evidence-based recommendations for the diagnosis of gout” [19], MSU crystal deposition is crucial evidence for gout diagnosis. In our study, DCS and tophi were frequently detected by US in gout patients, and they are notably increased with the disease history of gout. HAG was reported to be the most common sign at the early stage, especially in asymptomatic joints [20]. In an animal experiment where MSU was injected into the knees of rabbits, HAG was found frequently displayed in 75% of knees at an early stage (day 7 after MSU injection) [21]. Collectively, our data support that HAG is the US sign for early MSU crystal deposition, while DCS and tophi are a useful signature of MSU crystal deposition for chronic gout.

HAG was found to be associated with synovial lesions in this study. We found synovial lesion was notably detected at an early stage and gradually increased with the gout course extension. It has been reported that urate-lowering therapy (ULT) with febuxostat for 24 months reduced synovitis detected by MRI in patients with acute gout [22]. ULT may diminish HAG and improve synovitis and synovial hypertrophy. Bone erosion was known to be an irreversible injuriousness in gout [23]. In our study, tophi and DCS were both found to be correlated with bone erosion. Timely, ULT may minimize tophi and DCS and block bone injury in the gout patients.

Male, diabetes, obesity, low pH in urine, hyperuricosuria, and low urine volume have been reported to be the main etiologic factors of nephrolithiasis [24, 25]. Due to oversaturated urate and deposition of its crystals in the kidney, it is not surprising that a higher prevalence of nephrolithiasis is found in gout patients [26]. In this study, nephrolithiasis is remarkably relative to MSU crystal deposition in gouty arthritis. The result suggests that the patients with MSU crystal deposition in joints may be prone to suffer nephrolithiasis. Although MSU crystal deposition is related to high serum uric acid [27], systemic conditions such as genetic predisposition, geographical location, dietary indiscretion, and various metabolic characteristics should be considered as other risk factors [28, 29].

## 5. Conclusion

Ultrasound is a clinically convenient approach to detect MSU crystal deposits in joints for supporting the diagnosis of gout. HAG is considered an early sign of MSU crystal deposition in joints, whereas DCS and tophi correlate with bone erosion. Early ULT might be effective in the reduction of HAG and partially prevent synovitis and synovial hypertrophy. ULT should be considered when gout patients are detected with DCS or tophi in joints.

## Figures and Tables

**Table 1:** Demographics and clinical characteristics of the study population.

Variable	Gout	HUA	*p* value
Total number	202	43	—
Male/female	191/11	31/12	—
Age (years)	46.90 ± 14.82	44.47 ± 18.18	0.35
BMI (kg/m2)	25.59 ± 3.48	25.64 ± 3.03	0.93
SUA (*μ*mol/L)	524.24 ± 79.68	4	0.018^∗^
Ccr (mL/min)	95.55 ± 2.55	106.42 ± 5.54	<0.0001
TG (mmol/L)	2.91 ± 1.42	1.80 ± 1.29	0.0051^∗^
GLU (mmol/L)	5.73 ± 0.62	4.92 ± 1.68	0.0031^∗^
Cholesterol (mmol/L)	4.97 ± 1.04	4.70 ± 0.65	0.34

HUA: asymptomatic hyperuricemia; BMI: body mass index; SUA: serum uric acid; Ccr: creatinine clearance rate; TG: triglyceride; GLU: fasting blood glucose; ^∗^significantly difference (*p* < 0.05).

**Table 2:** Clinical characteristics of gouty attacks in the gout population.

Flared joint	Total	Left	Right	*p* value
MTP1	35.22% (187/531)	16.20% (86/531)	19.02% (101/531)	0.148
Ankle	29.19% (155/531)	13.93% (74/531)	15.25% (81/531)	0.496
Knee	18.83% (100/531)	9.04% (48/531)	9.79% (52/531)	0.671
Acrotarsium	8.47% (45/531)	4.71% (25/531)	3.76% (20/531)	0.399
Hands	3.95% (21/531)	1.88% (10/531)	2.07% (11/531)	1
Wrist	2.26% (12/531)	0.75% (4/531)	1.51% (8/531)	0.220
Elbow	2.07% (11/531)	0.94% (5/531)	1.13% (6/531)	1

MTP1: first metatarsophalangeal joint.

**Table:** (a) Global US findings in patients with gout and hyperuricemia

	Patients			
	Gout	HUA	*p* value	*X* ^2^

Total patient	202	43	—	—
Positive patient^∗^	154 (76.24%)	11 (25.58%)	<0.001	41.369

	Joints			
	Gout	HUA	*p* value	*X* ^2^

Total detected joints	1082	256	—	
Positive joint	358 (33.09%)	11 (4.30%)	<0.001	85.913
DCS	228 (21.07%)	6 (2.34%)	<0.001	50.320
HAG	64 (5.91%)	5 (1.95%)	0.04	4.168
Tophi	159 (14.70%)	0	<0.001	42.693

^∗^Positive patient: MSU crystal deposits in at least one joint of the patient.

**Table:** (b) US findings and clinical attacks in the joints of gouty patients

	Ever attacked	Never attacked
Total detected joints	487	595
Positive joints	277/487 (56.88%)	67/595 (11.26%)
DCS	159/487 (32.65%)	43/595 (7.23%)
HAG	38/487 (7.80%)	16/595 (2.69%)
Tophi	120/487 (24.64%)	16/595 (2.69%)

**Table:** (a) Synovial lesion and bone erosion in the patients with gout

	Joints
	Gout
Total detected joint	1082

Synovial lesion	192/1082 (17.74%)
DCS	47/192 (24.48%)
HAG	23/192 (11.98%)
Tophi	24/192 (12.50%)

Bone Erosion	82/1082 (7.58%)
DCS	46/82 (56.10%)
HAG	4/82 (7.69%)
Tophi	62/82 (75.61%)

**Table:** (b) Synovial lesion is related to HAG

Synovial lesion	Number	*p* value
DCS	47	0.385
HAG	23	0.01^∗^
Tophi	24	0.316

Pearson's chi-squared test, ^∗^significantly difference (*p* < 0.05).

**Table:** (c) Bone erosion is related to tophi and DCS

Bone Erosion	Number	*p* value
DCS	46	0.01^∗^
HAG	4	1
Tophi	62	0.001^∗^

Pearson's chi-squared test, ^∗^significantly difference (*p* < 0.05).

**Table:** (a) Clinical course and the MSU deposition in gout patients

	Course (years)				
	<1	1-5	5-10	10-15	>15
Patients	35	70	50	28	19
Total detected joints	159	380	283	150	110
Positive joints	13.84% (22/159)	20.79% (79/380)	39.93% (113/283)	48.00% (72/150)	65.45% (72/110)
DCS	6.29% (10/159)	12.89% (49/380)	23.32% (66/283)	33.33% (50/150)	48.18% (53/110)
HAG	5.03% (8/159)	4.74% (18/380)	8.48% (24/283)	4.67% (7/150)	6.36% (7/110)
Tophi	5.03% (8/159)	6.05% (23/380)	14.49% (41/283)	27.33% (41/150)	40.00% (44/110)
Synovial lesion	12.58% (20/159)	15.26% (58/380)	19.08% (54/283)	22.67% (34/150)	23.64% (26/110)
Bone erosion	0	2.11% (8/380)	6.36% (18/283)	18% (27/150)	26.36% (29/110)

**Table:** (b) US assessment in patients at first acute attack

Attacked joints [28].	MSU crystal deposition	
	Total	US signs
MTP1 [13]	8	3 DCS; 3 HAG; 2 tophi; 1 DCS + tophi
Ankle [6]	2	1 DCS; 1 HAG
Knee [1]	0	0
Acrotarsium [2]	1	1 HAG
Hand joints [2]	0	0

**Table 6:** Correlation of joint MSU deposition with nephrolithiasis.

Joint	Positive	Negative	Total
Kidney			
Positive	37	4	41
Negative	117	44	161
Total	154	48	404

Pearson's chi-squared test, *X*^2^ = 5.571, *p* = 0.018 (*p* < 0.05 considered as significantly difference).

## Data Availability

The datasets used and analyzed during the current study are available from the corresponding author on reasonable request.
